# Eriophyid Mites in Classical Biological Control of Weeds: Progress and Challenges

**DOI:** 10.3390/insects12060513

**Published:** 2021-06-01

**Authors:** Francesca Marini, Philip Weyl, Biljana Vidović, Radmila Petanović, Jeffrey Littlefield, Sauro Simoni, Enrico de Lillo, Massimo Cristofaro, Lincoln Smith

**Affiliations:** 1 Biotechnology and Biological Control Agency (BBCA), via Angelo Signorelli 105, 00123 Rome, Italy; massimo.cristofaro.cas@enea.it; 2 CABI, Rue des Grillons 1, 2800 Delémont, Switzerland; P.Weyl@cabi.org; 3 Department of Entomology and Agricultural Zoology, Faculty of Agriculture, University of Belgrade, Nemanjina 6, 11080 Belgrade, Serbia; magud@agrif.bg.ac.rs (B.V.); rpetanov@agrif.bg.ac.rs (R.P.); 4 Serbian Academy of Sciences and Arts, Knez Mihailova 35, 11000 Belgrade, Serbia; 5 Department of Land Resources and Environmental Sciences, Montana State University, Bozeman, MT 59717, USA; jeffreyl@montana.edu; 6 CREA Research Centre for Plant Protection and Certification, via di Lanciola 12a, 50125 Firenze, Italy; sauro.simoni@crea.gov.it; 7 Department of Plant, Soil and Food Sciences, University of Bari Aldo Moro, via Amendola 165/A, 70126 Bari, Italy; enrico.delillo@uniba.it; 8 ENEA Casaccia, SSPT-BIOAG-PROBIO, via Anguillarese 301, 00123 Rome, Italy; 9 USDA-ARS Western Regional Research Center, 800 Buchanan Street, Albany, CA 94710, USA; link.smith@usda.gov

**Keywords:** Eriophyidae, invasive alien plants, taxonomy, host plant specificity, risk assessment, impact, release, post-release monitoring

## Abstract

**Simple Summary:**

Eriophyid mites are tiny creatures, no bigger than a speck of dust. All species feed on plants and some can cause considerable damage. These mites have an intimate relationship with the plants that they live on, and most of the known species have been collected only from a single plant species, which suggests they are very specific to their host. They reproduce extremely quickly and can build up populations of millions, if not billions, of individuals within a single season. In recent years, research to evaluate their potential for the biological control of invasive plants has increased. Working with these minuscule herbivores poses challenges and offers opportunities for researchers. We review the most updated information in the context of weed biocontrol, giving current information on the challenges already faced and possible opportunities and solutions. We cover topics on taxonomy, evaluation of safety as biological control agents, impact and efficacy on the targeted plant species, and release and post-release monitoring. By offering the lessons learned from past research in a single updated document, our goal is to equip researchers with a valuable tool to help deal with the challenges and opportunities offered by eriophyid mites for the management of invasive plants.

**Abstract:**

A classical biological control agent is an exotic host-specific natural enemy, which is intentionally introduced to obtain long-term control of an alien invasive species. Among the arthropods considered for this role, eriophyid mites are likely to possess the main attributes required: host specificity, efficacy, and long-lasting effects. However, so far, only a few species have been approved for release. Due to their microscopic size and the general lack of knowledge regarding their biology and behavior, working with eriophyids is particularly challenging. Furthermore, mites disperse in wind, and little is known about biotic and abiotic constraints to their population growth. All these aspects pose challenges that, if not properly dealt with, can make it particularly difficult to evaluate eriophyids as prospective biological control agents and jeopardize the general success of control programs. We identified some of the critical aspects of working with eriophyids in classical biological control of weeds and focused on how they have been or may be addressed. In particular, we analyzed the importance of accurate mite identification, the difficulties faced in the evaluation of their host specificity, risk assessment of nontarget species, their impact on the weed, and the final steps of mite release and post-release monitoring.

## 1. Introduction

Over past decades, international trade and travel has increased exponentially, and with that also the spread of alien species [1]. Invasive plants (hereafter called weeds) can be defined as those species that are not native (i.e., alien) to the ecosystem under consideration, and that cause or are likely to cause economic or environmental harm or harm to human, animal, or plant health [2]. Invasive weeds cost billions of dollars annually in economic costs in addition to damages to ecosystem services and loss of biodiversity [3,4,5,6]. Conventional control strategies, including mechanical (e.g., mulching, tillage) and chemical (i.e., herbicides) methods, have long been used [7]; however, they are most cost-effective for intensively managed agroecosystems and sustainability should not be taken for granted [8,9]. Such methods are less practical and cost-effective to control invasive weeds on rangeland, forests and aquatic ecosystems, where there is growing interest in alternative strategies such as biological control [10].

Classical biological control consists of the importation and release of exotic host-specific natural enemies (biological control agents) to help reduce the density of the target weed and provide long-term control [11,12]. Many successes are reported in literature ([10,13,14,15,16] and references therein), which show clearly that this approach can be a cost-effective, environmentally benign and sustainable control method for invasive alien species [17,18,19].

Eriophyid mites (Eriophyidae) are among the smallest arthropods known (body length around 200 µm), which makes them difficult to study [20]. About 4800 species have been recognized, some of which are significant pests of agronomic plants [21], and many others (about 80% of those currently known) have been found in association with only one host plant [22], which implies that some species might be suitable to use as biological control agents [23]. Recently developed microscopic tools and molecular genetic methods have improved the ability of scientists to identify them, facilitating their study.

The purpose of this paper is to identify some of the critical aspects of working with eriophyid mites in classical biological control of weeds and to focus on how they have been or may be addressed. Aspects such as the importance of accurate mite identification, the challenge of evaluating host specificity, the risk assessment for nontarget species, and the impact on the weed, and the steps of mite release and post-release monitoring are discussed using pertinent examples.

## 2. Classical Biological Control of Weeds Using Eriophyid Mites

**Figure 1:**
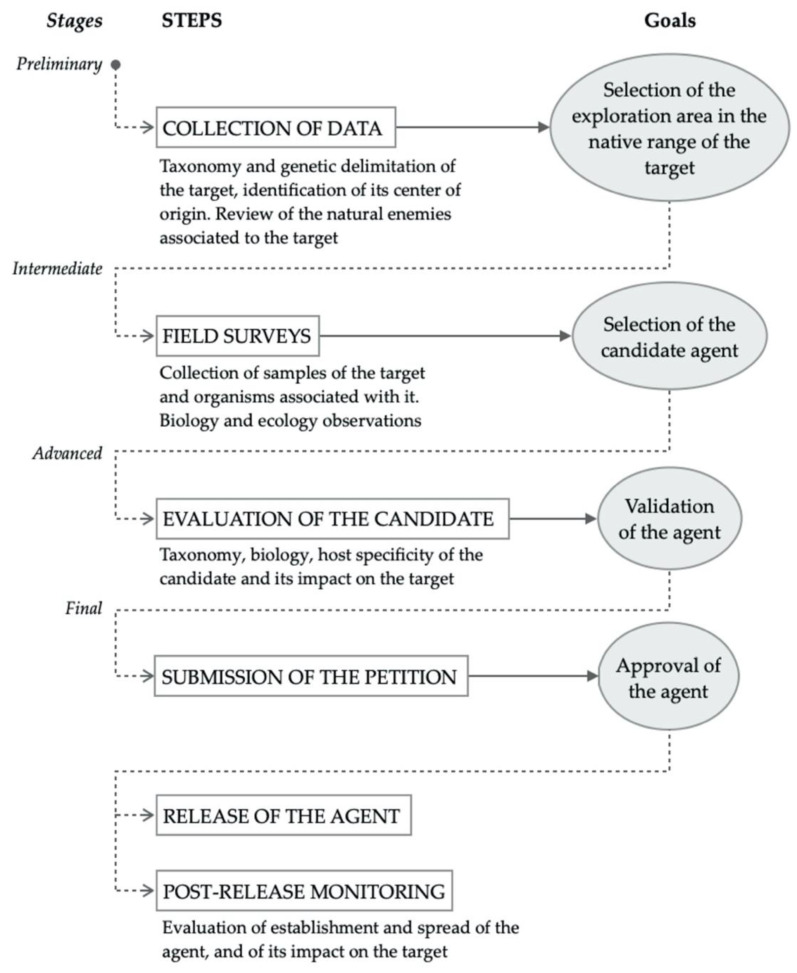
Main stages, key steps, and primary goals of a weed classical biological control program, including some essential activities for each step.

In general, a classical biological control program of weeds consists of a progression of several steps, such as literature search and field surveys, evaluation of the candidate selected, submission of a petition to obtain governmental approval for its release, release, and post-release monitoring of the agent (Figure 1) [12].

Recently, interest in the use of eriophyid mites as prospective candidates in classical biological control programs of weeds has increased, since they possess many attributes that are likely to favor them as potential biological control agents. These include an intimate and coevolved relationship with their host and high host specificity, high reproductive rates with very short generation times, dispersal by wind, and potentially high impact on target plants [21,24].

By feeding, eriophyid mites may induce significant disturbances in plant morphology, including stunting of leaves, reductions in internode length and in the production of fruit and seeds. In some cases, they may cause reduced growth in root, as well as in above ground biomass and reproduction [24,25]. Despite those desirable features, only few eriophyid mite species have been intentionally released. The earliest species successfully released date from the 1970s and 1980s, and they are *Aceria malherbae* Nuzzaci, *Aceria chondrillae* (Canestrini), and *Aculus hyperici* (Liro) ([23] and references therein). Most of the eriophyid mite species that have played some role as biological control agent thus far are actually adventive, i.e., exotic organisms which were accidentally moved, or spread naturally to another country. Some others were intentionally redistributed, and only a few of them were effectively evaluated and gained regulatory approvals for their intentional introduction (Table 1).

In the context of classical biological control of weeds, working with eriophyid mites may be particularly difficult, starting from the lack of knowledge regarding their biology, ecology, and behavior. Due to their microscopic size and tendency to hide within plant structures (e.g., galls, various deformations or common protective organs), the handling and rearing of eriophyid mites is particularly challenging, as is making any direct observations on them [21]. Moreover, some eriophyid mites are highly coevolved with their host plant, and they may be ineffective biological control agents because of reduced harmfulness of the mite and/or high tolerance of the plant [23]. Susceptibility of eriophyids to biotic (e.g., host plant resistance and natural enemies) and abiotic (e.g., soil composition, rain, wind and climate) factors may also prevent them from achieving the densities necessary to reduce host plant populations, and thus impact the target weed [23,26] (for examples see limiting factors in Table 1).

## 3. Modern Taxonomy and Its Role in Improving Classical Biological Control Programs

Building on the traditional morphological approach to the identification and/or description of an eriophyid mite, new trends appeared in the last decade, paving the way towards integrative taxonomy [37]. Nowadays, methods of linear and geometric morphological analysis are applied more frequently, since they allow studying intraspecific variation, including host-adapted strains, host-races, or even cryptic species. These phenomena are particularly common in eriophyid mites because of the intimate association with their hosts, the lack of long-distance host seeking ability, and high reproductive rates [38,39]. Molecular genetic tools, which have begun to be employed in eriophyid mite studies [40], should further advance mite taxonomy. In particular, a complex approach using the combined techniques of phase-contrast light microscopy, diffraction interference contrast microscopy, confocal laser scanning microscopy (CLSM), and scanning electron microscopy (SEM), as well as sequencing of standard DNA regions, including the mitochondrial gene cytochrome oxidase subunit I (mtCOI) and nuclear regions such as the internal transcribed spacers ITS1 and ITS2, supports the description of eriophyid mites with much more detail, much of which is also quantifiable, than before [40,41].

An integrative approach can help in establishing reliable criteria to determine species in Eriophyoidea. Most papers published today concerning alpha–taxonomy are prepared following traditional methods, but there are increasing numbers of species descriptions employing methods of traditional morphological taxonomy combined with the support of DNA sequences of one (typically mtCOI) or a few genetic regions [42,43,44,45]. For example, the newly recognized species *Aceria artemisiifoliae* Vidović & Petanović, a potential biological control agent of common ragweed (*Ambrosia artemisiifolia* L., Asteraceae), was described following both morphological and bio-molecular approaches [46].

The study of intraspecific phenetic and genetic variability in order to verify the current status of taxa began as early as the second half of 20th century, and the intraspecific variability of selected taxa was the subject of interest of some authors in the following decades. A quantitative approach with sample selection of adequate size and the use of restrictive statistical tests (i.e., multivariate analyses based on at least 30 individuals and more than 20 characters) was initiated by Skoracka et al. [47], followed by similar studies using linear and geometric morphometry to establish phenetic similarities or differences [48,49,50,51,52]. At the end of the first decade of the new century, such data began to be combined with molecular genetic analyses of the barcode region in order to obtain congruence with phenetics [44,53,54].

Since the beginning of the last decade, the integrative approach to taxonomy of eriophyid mites has also been applied to the description of new candidates for the biological control of weeds. For example, studies on complexes of mite populations occurring sympatrically on closely related host plants have contributed to the discovery of some cryptic species of the genus *Aceria*, such as those within *Aceria anthocoptes* (Nalepa) complex (i.e., *A. anthocoptes* s.s. ex *Cirsium arvense*; *A.* cf. *anthocoptes* ex *C. heterophyllum*; *A.* cf. *anthocoptes* ex *C. eriophorum* and *A.* cf. *anthocoptes* ex *C. creticum*, with genetic divergence ranging from 11% to 18.1% [55]) or the four cryptic species from *Aceria drabae* (Nalepa) complex (i.e., *Aceria auriniae* n.sp. ex *Aurinia petraea*, *Aceria berteroae* n.sp. ex *Berteroa incana* and *Aceria sisymbrii* n.sp. ex *Sisymbrium orientale*, with genetic divergence ranging from 14.4% to 25.9% [56]).

Studies of Eurasian populations of some species belonging to the genera *Aculodes* and *Metaculus* are currently ongoing. In particular, after the description of *Aculodes altamurgiensis* de Lillo & Vidović from medusahead (*Taeniatherum caput-medusae* (L.) Nevski, Poaceae) [57], a morphologically similar mite was found on cheatgrass (*Bromus tectorum* L., Poaceae), occurring sympatrically with *A. altamurgiensis* on medusahead. However, the quantitative morphometric analysis showed divergence between the two mites, which was also confirmed by measuring the genetic divergence (mtCOI distance = 18%). Thus, by using an integrative approach it was possible to determine that the two mite populations occurring on medusahead and cheatgrass, respectively, are actually two distinct species [58].

The phenomenon of cryptic speciation was also recorded in mites of the genus *Metaculus*, some of which have been evaluated as biological control agents of some Brassicaceae. To date, three *Metaculus* species have been recorded, i.e., *M. lepidifolii* Monfreda & de Lillo ex *Lepidium latifolium*, *M. rapistri* Carmona ex *Rapistrum rugosum* and *M. diplotaxi* Petanović & Vidović ex *Diplotaxis tenuifolia* [45,59,60]. *Metaculus rapistri* was initially described by Carmona (1969) from samples of *R. rugosum* collected in Portugal. Thereafter, a supplementary morphological description of this species was provided by Monfreda and de Lillo [60] from samples of *Isatis tinctoria* L. collected in Turkey. In 2017, *M. rapistri* ex *I. tinctoria* was recorded at two more localities, in Italy and Germany. Further research of quantitative morphometric characteristics and analysis of mtCOI sequences showed the existence of cryptic species within the *M. rapistri* complex, and *M. rapistri* ex *R. rugosum* is a distinctly different species than *Metaculus* sp. ex *I. tinctoria*. Furthermore, the *Metaculus* sp. ex *I. tinctoria* population from Turkey is different from the specimens from Germany and Italy, which indicates the existence of two cryptic species adapted to the same host plant [61,62].

In morphological and molecular investigations on eriophyid mites from Russian olive (*Elaeagnus angustifolia* L., Elaeagnaceae) no differences were observed among *Aceria angustifoliae* Denizhan et al. populations collected at various sites in Eurasia (i.e., Serbia, Iran, Uzbekistan and Armenia) suggesting a single species [63].The morphological comparison of these populations with those found on Russian olive in the USA (i.e., Wyoming, Colorado, and Montana) suggested the existence of *A. angustifoliae* in the USA. However, molecular analysis showed that, although the American populations belonged to the genus *Aceria*, they were genetically different from *A. angustifoliae* in Eurasia. Molecular comparisons with eriophyid mites collected from other Elaeagnaceae, resulted in a match with an undescribed species collected from the North American native *E. commutata* L. These data mean that *A. angustifoliae* occurring on Russian olive of Palearctic origin is not yet recorded in the USA, however, morphologically these may be considered cryptic species with those on *E. commutata*, which has implications for future monitoring of biological control of Russian olive in North America [30].

The modern taxonomic approach was also important for the identification of *Aceria acroptiloni* Shevchenko & Kovalev. When this species was described, three morphs were observed on Russian knapweed (*Rhaponticum repens* (L.) Hidalgo, Asteraceae) [64]. Based on morphological investigation using conventional light microscopy and a more precise CLS microscopy, it was possible to determine that these morphs were three different mite species, all occurring on the same host plant [65].

Another example of the usefulness of the integrative taxonomy approach is the resolution of the identification of at least two of the four eriophyid mite species recorded on tree of heaven (*Ailanthus altissima* (Mill.) Swingle, Simaroubaceae), i.e., *Aculops taihangensis* Hong & Xue and *Aculus mosoniensis* (Ripka). Based on morphological observations, de Lillo et al. [66] suggested that *A. taihangensis* and *Ac. mosoniensis* were likely synonymous, and the subsequent molecular comparison showed no significant variation in nuclear region ITS1 (*p*-distance = 0.02 ± 0.02 bp) confirming the synonymy of the two species [67]. The same approach could help solve the identification of the other two species recorded on tree of heaven, i.e., *A. ailanthi* Lin, Jin, & Kuang, and *Ac. altissimae* Xue & Hong. In fact, according to Amrine J., *Ac. altissimae* may be a junior synonym of *A. ailanthi*, because the morphological differences between the species are slight and may be attributable to procedural-artifacts (e.g., over clearing specimens prior to slide mounting, differences in interpretation of characters of slide mounted specimens, poorly made illustrations) [68]. This last point raises one more issue. Different authors can use different slide mounting methods, and this may affect the comparison of the descriptions. Overclearing, underclearing, body stressing, flattening, squashing, imprecisions in measurements, microscopy details, and experience of the operators: all these aspects may lead to mistakenly perceived differences among specimens observed by different scientists. Therefore, it is important to use standardized protocols for morphological observations to minimize such discrepancies [69].

In contrast to the previously mentioned examples is the case of *Aceria alhagi* Vidović & Kamali, a promising candidate for the control of camelthorn (*Alhagi maurorum* Medik., Fabaceae) [70]. Investigations of genetic variability by molecular analyses (mtCOI) among populations from Iran, Turkey and Armenia showed genetic divergence ranging from 0.0 to 0.6%, suggesting that they represent one species [71]. These data suggest that some eriophyid species appear to be very uniform over wide geographic areas, whereas some others show a wide intraspecific phenetic and genetic variability. For example, the study of *A. anthocoptes* s.s. ex *C. arvense* populations from different regions showed that the geographical factor may have an impact on both phenetic and genetic variability [51,55]. Landmark-based geometric morphometric methods to study morphological variability of three body regions (ventral, coxigenital and prodorsal) revealed significant differences between *A. anthocoptes* s.s. inhabiting European (Serbia) and North American (Colorado) *C. arvense*. Moreover, European populations of *A. anthocoptes* s.s. from *C. arvense* are characterized by higher inter-population size and shape variability than their North American counterparts [55]. Finally, molecular comparisons of seven populations from different localities in Serbia showed a genetic divergence ranging from 5.6% to 6.2% [59]. There is still debate around the threshold (i.e., the percentage of genetic divergence) for distinguishing eriophyid species on the basis of COI gene. The first report was presented by Skoracka and Dabert [72], who showed that reproductively incompatible strains of *Abacarus hystrix* exhibit more than 20% sequence divergence in the COI gene and 0.2% sequence divergence in the nuclear D2 region of 28S rDNA. A barcoding gap analysis identified the gap for within- and between-species to be 13 to 15% for COI sequences within the *Abacarus histrix* s.l. complex [73]. Studies of other taxa have reported a range of interspecific and intraspecific distances, but without a gap analysis (e.g., [53,57,74,75]).

## 4. Evaluation of Eriophyid Mite Host Plant Specificity, and Its Implications

Eriophyid mites are considered to be often highly host-specific, but it should not be forgotten that in the past their host ranges have been mainly deduced on the basis of collection records often based on few samples, instead of being determined by quantitative field data or experimentation [22,76]. The increasing interest in the use of eriophyid mites as agents for the biological control of weeds, as well as the need to control agronomic pest species (e.g., *Aceria tosichella* Keifer, *Cecidophyopsis ribis* (Westwood), *Aceria cajani* Channabasavanna [77,78,79]), has led scientists to increase the effort to better understand their host plant interaction and potential host range and, resulting in significant improvements of the host-specificity screening methods.

Since the beginning of regulated biological control, laboratory tests have been used to distinguish those nontarget plant species that are clearly not suitable hosts for the candidate mite agent, even though they may be closely related to the target species [80]. These bioassays measure parameters such as adult feeding, oviposition and larval development of the prospective candidate on various host species and are often carried out in confined spaces and under artificial conditions and may produce results that differ from natural behavior in the field. On the other hand, open-field tests imply the total absence of artificial barriers (e.g., screens, cages, tubes, etc.) to the natural movement of the agent, and to its natural enemies, as well as to the exposure to natural environmental conditions. Therefore, under field conditions, the agent can show its natural behavior and exercise a free choice [81,82]. Eriophyid mites are mainly dispersed by wind ([39,83,84,85,86] and references therein) and hence have limited chance to depart from unsuitable plants under laboratory conditions (e.g., for lack of air movement or physical contact between different host plants), which may increase the tendency of the mites to probe and/or feed on nontarget plants. Consequently, laboratory experiments with eriophyid mites are generally no-choice (starvation) tests, which may delineate the fundamental (or physiological) host range. However, this may yield ‘false-positive’ results and overestimate the risk of attack under field conditions, so the results obtained by them should be interpreted cautiously [80,87]. Conducting host specificity tests in the field may be the best way to assess the realized (or ecological) host range, which is usually a sub-set of the fundamental one [82], and to validate the results obtained by laboratory experiments.

During the past 30 years, the study of eriophyids in field experiments has significantly increased [22], and several species have been shown to be host specific in the field, even though they were observed to develop on and damage some nontarget plants under laboratory or greenhouse conditions (e.g., *A. hyperici* and *A. malherbae* biological control agents of St. John’s wort (*Hypericum perforatum* L., Hypericaceae) and field bindweed (*Convolvulus arvensis* L., Convolvulaceae), respectively; *Aceria centaureae* (Nalepa), a candidate agent of diffuse knapweed (*Centaurea diffusa* Lam., Asteraceae) ([23] and references therein)). For example, *Aceria solstitialis* de Lillo, Cristofaro et Kashefi, a candidate for the control of yellow starthistle (*Ce. solstitialis* L., Asteraceae), showed a wider host range under artificial than in field conditions [88]. The mite was observed to persist (i.e., the plants remained infested with live mites) for as long as 60 days on some nontarget plants under laboratory conditions (i.e., *Ce. diffusa*; *Ce. cyanus* L., *Carthamus tinctorius* L. and *Cynara scolymus* L.), whereas in the field it was not found on most of nontarget species tested (mites occurred only on *Ce. solstitialis* and *Ce. cyanus*). Similar results were observed also for *Aceria salsolae* de Lillo & Sobhian, a candidate agent against Russian thistle (*Salsola tragus* L., Chenopodiaceae) [31,89,90]. In laboratory experiments *A. salsolae* produced small populations on some nontarget species (i.e., *Atriplex coronata* S. Watson, *Bassia hyssopifolia* (Pallas) Kuntze, *B. prostrata* (L.) A.J. Scott, *Kochia scoparia* (L.) Schrader, and *Suaeda calceoliformis* (Hook.)), whereas in the field it was able to persist only on one of them (i.e., *A. coronata*) and at extremely low densities, with no evidence of reproduction (i.e., no juveniles were found) [31].

Eriophyid mites represent a potential risk for nontarget species if they are able to at least persist (i.e., survive) on these plants. Life expectancy of eriophyid mites is still poorly known. Although 4 to 5 weeks has been reported for non-diapausing females (i.e., the protogyne morph) [91], eriophyid mites can survive even longer under cool conditions, especially if they are protected from desiccation and can obtain some nutrition. In a recent study on persistence of five eriophyid species in water droplets, mites survived for up to 1 to 11 days at 25 °C, depending on species and morph, and up to 1 to 7 weeks at 5 °C [92]. *Aceria tulipae* (Keifer, 1938), which today would be identified as *A. tosichella* [93], survived at least 80 days on potato dextrose agar (being tested as an artificial substrate), but they did not oviposit until they were transferred to wheat plants [94]. In order to estimate eriophyid survivorship, it is important to monitor through time the mite population on the plants. For example, during the first host specificity field experiment with *Ac. mosoniensis*, by performing more than one sampling, it was found that the mite can persist for at least one month on two nontarget species among those tested. In particular, at 34 days after the inoculation of about 15 individuals on each plant, a few live mites were recorded on the nontarget hosts *Juglans regia* L. and *Quercus ilex* L. (i.e., 11 and 8, respectively) compared to thousands of live *Ac. mosoniensis* collected from its natural host, tree of heaven (i.e., 9218). At 47 days post-inoculation, no live mites were found on any of the nontarget plants (only 3 and 1 dead mite were remaining on *J. regia* and *Q. ilex*, respectively), while on tree of heaven the number of live mites further increased (i.e., 16,868). A second experiment determined that *Ac. mosoniensis* can survive even longer (i.e., up to two months) on some other nontarget species. In particular, live mites were found on *Olea europaea* L. and *Rhus coriaria* L. up to 63-days from the inoculation (3 and 1 live mite, respectively, compared to 5263 live *Ac. mosoniensis* collected from tree of heaven) [M. Cristofaro, unpubl. data]. The determination of the status of ‘dead’ or ‘alive’ of the specimens found on the plants is hence crucial for the estimation of the eriophyid mite survival. Moreover, since some species can be particularly long-lived even in suboptimal conditions, caution should be taken in choosing the duration of a host specificity experiment. Finally, due to their very small size, the identification of eriophyid mite species, whether by morphological or molecular methods, cannot be achieved without killing the individuals. This means that dead and live specimens have to be recorded and stored separately at the time of collection, because it is not possible to determine their status from preserved or mounted specimens.

The presence of live mites on nontarget plants does not necessarily mean they were able to survive on those plants. It is not unusual that mites found on nontarget plants during field experiments may have recently dispersed from nearby heavily-infested target plants [23,26,88]. Aerial dispersal is widely considered the main mode of dispersal for eriophyid mites [39,86,95], and mites presumably have little or no control over where they land, so behavioral selectivity might involve assessing the plant and either staying to feed or dispersing by the next available wind [96]. Therefore, it is important to distinguish between ‘transitory’ live mites found on nontarget plants and signs of infestation. During field surveys of *A. acroptiloni* in Iran, very low densities of the mite were recorded on several nontarget plants (i.e., *Onopordum* sp., *Carthamus* sp., *C. lanatus* L., *C. oxyacantha* M.Bieb., *Centaurea squarrosa* Willd., *Lactuca serriola* L.), even though there was no evidence of symptoms due to feeding activity. By using water pan traps to detect aerially dispersing mites [86], it was concluded that the few *A. acroptiloni* recorded were actually mites randomly dispersing and could be considered transitory visitors [97].

The level of injuries caused by arthropods attacking a plant generally depends on the density of individuals present [98], unless other factors are also involved (e.g., plant pathogens, stressful climatic conditions, etc.). Therefore, in the case of a prospective biological control agent that persists on a nontarget plant, it is particularly important to determine if it can multiply enough to achieve population densities that impact the plant [18]. Assessing the population structure (i.e., the number of eggs, nymphs and adults, and also of males and females) after suitable time can help to determine if the mite is able to reproduce and develop a population on the plant, and how big this could become, especially if the starting number of mites inoculated is known. During a laboratory no-choice study, *A. salsolae* was observed to persist for up to 5 weeks and even reproduce on one of the nontarget species, *A. coronata*, but it never attained populations anywhere close to those that developed on *S. tragus* [31]. However, in the field the mite could persist for up to 9 weeks, but the absence of juveniles indicated that no reproduction occurred. These data suggest that even though *A. salsolae* could persist for a long time on *A. coronata*, the mite did not reproduce on it under natural field conditions. Thus, as in the case of *Ac. mosoniensis*, some plants may be suitable enough to support survivorship but not necessarily reproduction of the eriophyids and hence are not likely to be harmed by the mites.

Ability to reproduce on nontarget species does not necessarily mean that mites will cause significant damage. For example, although able to develop on some nontarget plants in pre-release studies, *A. hyperici* was released in Australia as a biological control agent against St. John’s wort (Table 1). The mite survived and reproduced on at least four nontarget species, including a species native to Australia (*Hypericum gramineum* G.Forst.) [24]; however, it had negligible impact on all measured indices of growth and reproduction of this plant in the field and therefore was not considered harmful [99]. On the other hand, even the induction of plant injuries is not necessarily a sign of successful mite reproduction [100,101]. This is the case of *A. malherbae*, which was released in the USA and South Africa against field bindweed (Table 1), even though it caused galling on three *Convolvulus* and 12 *Calystegia* species in laboratory and screen house studies [100,101,102,103]. In laboratory studies, galling, but not reproduction, was observed on the *Calystegia* species while both occurred on field bindweed [100,101]. In a field experiment, galling was observed on several nontarget species during the summer in which mites were inoculated, but not the following year, suggesting either failure of reproduction and/or survival of *A. malherbae* through the winter (which normally occurs underground on the roots), whereas field bindweed was galled the following year (Hansen, R.W. pers. comm. in [23]). These data suggested that mite requirements for reproduction are more restrictive than for gall induction [100]. In the field, both *A. malherbae* and *A. hyperici* have not had significant impact on nontarget species that are known to be within their physiological host range. Thus, in cases where a nontarget species is attacked under no-choice conditions (i.e., laboratory conditions), assessing the risk of attack under more natural conditions may provide convincing evidence of the safety of the candidate as biological control agent [87]. Furthermore, as shown in the cases of *A. hyperici* on *H. gramineum* [99] and *A. salsolae* on *A. coronata* [31], it is important to quantify any reduction in fitness (size, survivorship or reproduction) of the nontarget plant by the agent [28].

The kind and severity of the induced symptoms depend on the host-mite specificity and mite density, but also on the organs infested, phenology and physiological conditions of the plant. Therefore, in order to produce results that accurately predict risk of damage to nontarget plants in the field, experiments have to be designed based on knowledge of the life history of both the eriophyid mites and plants. Plants have to be at a suitable developmental stage, with plant structures vulnerable to the mites. Plants that do not have tissue at the right stage for inducing galls may mask susceptibility, whereas those that have softer tissue due to the artificial growing conditions could be more susceptible than normal.

Another critical aspect of the risk assessment for nontarget species to be attacked by a prospective biological control agent is the duration of experiments to ensure that any possible injury would be observed. Marini et al. [31] inoculated *A. salsolae* on test plants when they were little bigger than seedlings, providing to the mites tender tissues on which they could feed, and ended the experiment when each host species reached the mature growth stage (i.e., fruits). This approach allowed measuring the development of the mite population (as discussed above) and also recording and quantifying any potential impact on the plants, following both mites and plants for the whole season.

The use of eriophyid mites in the biological control of weeds also implies the possibility of dealing with biotypes of the target that are resistant or less susceptible to the mite selected as agent for its control [104]. Variability in the susceptibility of the target weed to its agent also should be considered as variability in the performance of the eriophyid on its target, in terms of colonization rate and impact, and hence in its efficacy against the target. This means that to ensure the introduction of an appropriate agent, and the success of its establishment, the phenotypic structure of the target weed should also be studied. In fact, the variability in the susceptibility of plants to mites is not uncommon, especially for those plant species which present different forms [24].

*Aceria cynodoniensis* (Sayed) is the best example of an extremely specific species, which can only develop on particular strains of Bermuda grass (*Cynodon dactylon* (L.) Pers., Poaceae) parentage, but not on hybrids [105]. Another example is *A. chondrillae*, whose populations from different regions are specialized on the corresponding forms of rush skeletonweed (*Chondrilla juncea* L., Asteraceae) from those same regions [106]. The plant has at least four different genotypes that vary in resistance to the mite [107,108]. The Greek mite strain was found to be highly effective against the predominant narrow-leaf form of rush skeletonweed in Australia, but it had low or no impact on the other forms of the weed present in Australia or in North America. On the other hand, the Italian mite strain induced galling on the North American forms of rush skeletonweed, but not on the most common form in Australia [24,107,108]. A similar pattern was observed for *Floracarus perrepae* Knihinicki & Boczek. Extensive samplings, genetic analyses and laboratory tests revealed location-specific haplotypes of the mite and its host plant, Old World climbing fern (*Lygodium microphyllum* (Cav.) R. Br., Lygodiaceae), across the native distribution, and in particular that *F. perrepae* populations from various locations were best able to induce galls on the local fern haplotype [109]. Thus, both *A. chondrillae* and *F. perrepae* are examples of a high degree of specialization on different host-plant forms (i.e., host-adapted strains). In the case of *A. hyperici*, the plant has populations completely resistant to the mite. In particular, among the different populations of St. John’s wort present in Australia, two of them did not support the development of *A. hyperici* populations, while the other four were susceptible, but showed some variations in population growth and impact on the plants [24]. This pattern is similar to what was also observed with *A. malherbae* on field bindweed, *Aceria lantanae* (Cook) on lantana (*Lantana camara* L., Verbenaceae) or *A. altamurgensis* on medusahead, for which variations in the susceptibility to infestation was experimentally demonstrated by testing different populations of their respective targets [103,110,111,112]. However, *A. altamurgensis* performed very poorly on the medusahead plants from the location where it was collected (i.e., Apulia, Italy), whereas it performed much better on plants from Sicily, Italy, and from Idaho and Nevada, USA [110]. Generalizing, these examples point out the importance of including multiple native- and invaded-range populations of the target weed in pre-release evaluations, but also to test additional mite strains to control resistant varieties of the target plant.

## 5. Evaluation of Eriophyid Mite Impact on the Target Weed, and Its Implications

Predicting and measuring impact of weed biological control agents is a challenging task and typically fewer resources are channeled into pre-release studies when compared to safety studies [98,113]. However, nowadays these studies are increasingly being conducted, and not only to reduce the probability of releasing an ineffective agent [114], but also, to better understand why certain biological control programs are a success while others fail [115,116].

To date, the majority of eriophyid mites released have not had the expected impact that pre-release studies have predicted, but rather impact is typically variable across the landscape [28,115], as turned out for *A. malherbae*. Laboratory studies indicated that the mite would be likely to achieve significant reductions in both shoot (37%) and root biomass (50%) of field bindweed [117], but this has not been the case throughout the range of field sites [23,118]. There is much speculation on the differences observed in the outcomes at the different sites; however, there appears to be a clear link between climatic variables and chances of success, with drier and warmer years resulting in a greater probability of success, with some sites having as much as 95% reduction in field bindweed [23]. Interestingly, particular land use patterns, such as mowing and herbicide application at sub-lethal doses, appear to be able to be integrated into the biological control program increasing the impact of this mite and success of the program in general [117,119]. This is not unique to this system and mowing has been investigated as an integrated management strategy for the control of the light pink 163LP variety of lantana using *A. lantanae* [120]. In particular, the combination of mowing did not seem to affect the mite’s occurrence and infestation patterns [111], however, there was a 78.5% increase in the number of galled inflorescences per shoot, when compared to plants that were not mowed [120]. These studies highlight potential problems of relying solely on laboratory studies. There are indeed many abiotic (e.g., climate, soil, wind, etc.) and biotic (e.g., pathogens, predators, host plant resistance, etc.) interactions which may limit the eriophyid mite impact once they are released in the field [23,26]. Thus, it is particularly important to gather information in the native and non-native range on potential limiting factors, not only climate, but also what effect particular land use patterns may have on mite populations and how these may influence the impact of the mite.

Field studies through observations and experiments have shown not only massive reductions in seed set of individual plants (e.g., 66% by *A. angustifoliae* against Russian olive [121], to 95% by *A. alhagi* against camelthorn [70], and up to 98% by *A. acroptiloni* against Russian knapweed [122]), but also, impressive reductions in biomass (e.g., 66% reduction in size of Russian thistle by *A. salsolae* [89] and a 49% reduction in above ground Old World climbing fern biomass by *F. perrepae* [123]). Although these results are encouraging, many of these experiments are conducted in the best possible conditions for establishment and rapid buildup of the mite population on plants. This can be achieved by early season inoculations to boost the population enough to achieve impact, or attempting several different methods of establishment in order to obtain the best results [122]. Moreover, experimental set ups are usually short-term (1–2 years) and at individual plant level. For example, the only feasible way to obtain an impact assessment of *A. angustifoliae*, although establishment was successful, was to collect data from existing infestations under natural conditions, by comparing branches infested with mites with those that were free of mite attack [121]. This resulted in an estimated 2/3 reduction in seed set on infested branches by not only affecting fruit production directly, but by also reducing the length of fruit bearing branches, further compounding the impact [121]. Although this suggests a high level of impact and will likely slow the invasion potential of Russian olive in North America, there are certain limitations linked to observational data. Such as, it is unclear whether the mites are present and impactful on certain branches or trees that are inherently unlikely to produce many fruits. By selecting for mite infested branches on particular trees, it is possible that the impact may be overestimated, and thus, difficult to translate the individual branch level to the population level.

Factors that limit or influence eriophyid mites under field conditions may be so subtle that they can be difficult to detect or understand. To cite an instance, after a successful impact experiment with *A. acroptiloni* in Shirvan, northern Iran [122], due to logistical reasons, the host range experiments were moved to Mashhad, about 200 km to the south-east [26], but establishment on the control plants failed despite trying different techniques for six years [124]. In this case there was a slight altitudinal drop from 1100 m to 980 m above sea level, however, no quantifiable differences were detected comparing several abiotic factors, such as soil structure and type as well as a suite of climatic variables [26]. Due to this, it was decided to suspend any further work with this species, since the limiting factors which contributed to the failed establishment after moving just 200 km were not identified, it is unlikely that *A. acroptiloni* will establish and successfully control Russian knapweed in North America [124].

In the case of *F. perrepae*, pre-release impact experiments revealed that the mite was capable of a significant reduction in above ground (by 49%) and below ground (by 35%) Old World climbing fern biomass [123], however, this level of impact is yet to be observed in the field. A likely explanation of the limited impact could be linked to host plant susceptibility (also discussed above) [125] however, predators and pathogens known to impact mite populations [123] cannot be completely ruled out. Although predators and pathogens were observed to reduce the mite populations, there appeared to be little or no measurable effect on the impact of *F. perrepae* [123]. In a recent study, David et al. [126] identified wind speed to be positively associated with mite densities and suggested it may be directly linked to within and between site dispersal, ultimately influencing the probability of establishment and impact. Another study showed how the time of year and degree of shading could affect the impact of this mite on the growth of its host plant [127].

The identification of the limiting factors that are linked to a reduced impact is a challenging task, especially because sometimes they may not be obvious and/or rather related to the basic biology and behavior of eriophyids.

## 6. Release and Post-Release Monitoring of Eriophyid Mites: Last but Not Least Steps of Classical Biological Control Programs

Release and distribution of eriophyids for biological control of weeds generally follow after a progression of overseas collection, quarantine screening, establishment of rearing colonies, and initial releases leading to field establishment from which redistributions can be made ([128]). Although seemingly straight forward, the flow of agents may be hindered by procedural problems such as regulatory impediments, collection difficulties, and shipping delays, along with biological or ecological challenges that may pertain to each particular eriophyid mite. Overseas collections may be limited by international conventions, such as complying with requirements of the Nagoya Protocol on the Access to Genetic Resources and the Fair and Equitable Sharing of Benefits Arising from their Utilization, Convention on Biological Diversity [129,130]. Collections of agents or specific mite genotypes from some countries may be limited or impossible. For example, the collection and shipment of *M. lepidifolii* for perennial pepperweed (*L. latifolium* L., Brassicaceae) from Turkey was hampered by the difficulty of obtaining an export permit for the mite and by the political climate of that time in Turkey [131] As a result, surveys and subsequent collections of *M. lepidifolii* were moved from Turkey to Kazakhstan, from where export permits were obtainable [128]. A similar situation occurred with *A. angustifoliae* for which collections and experimental evaluations were moved from Iran and/or Turkey to Serbia, where the mite is also present [132].

Once regulatory approval for release is granted, the strategies for release and monitoring of eriophyid mites are similar to those of other arthropod agents utilized in classical biological control. However, due to their small size and ability to build to high populations, eriophyid mites could also lend themselves, like plant pathogens, to an inundative or bioherbicidal approach [133]. While nearly all eriophyid mites released for invasive weed management have been used in the classical approach, inundative methods should not be overlooked as an option in appropriate situations. Compared to insects, eriophyid mites are very small and are thus difficult to release as individuals and are generally released as a collective along with their host plant or infested plant tissue. For example, *A. malherbae* is distributed in Colorado by pulling or cutting infested field bindweed plants and placing the vegetation in paper bags which are given to the public, to be opened for release on their property [134]. The numbers of individuals available for initial releases from containment laboratories are usually limited. Prior to release into the environment, eriophyid agents have to be processed in a containment facility to screen out potential natural enemies such as phytoseiid mites, fungal pathogens of eriophyids, and other unwanted organisms such as other species of eriophyids, thrips, aphids, spider mites, etc. [135,136,137,138]. Mite identity is critical, especially on plant species that may harbor multiple eriophyid species, such as Scotch broom (*Cytisus scoparius* (L.) Link, Fabaceae) (two mite species, i.e., *Aceria genistae* (Nalepa) and *Aceria davidmansoni* Xue, Han & Zhang) [139], and Russian knapweed (three mite species, as discussed above) [65,97,122]. Eriophyid mites are known vectors of plant pathogens, especially viruses or suspected viral agents [140]. Thus, there was concern about the risk of *Cecidophyes rouhollahi* Craemer, a prospective agent for cleavers *(Galium* spp.), because a related gall mite which attacks *G. aparine* L. was thought to be associated with a plant virus [141]. Prior to release, mites were tested for 13 viral groups using PCR, with a second test conducted for false positives [142]. To further reduce the risk of accidental introduction of unwanted organisms, importations of eriophyid mites to containment facilities are limited in number. For example, for *A. malherbae* only one shipment was received and released in Texas, USA [143]; in Montana, USA, *A. malherbae* was imported for three years [144]; and two importations of *C. rouhollahi* were made in Canada [142]. For *F. perrepae*, only one importation was made from Australia to Florida, USA [125].

To initiate a rearing colony, a clean colony protocol of transferring individual mites to clean host plants is utilized. This protocol was employed with the broom gall mite, *A. genistae* [145] and with other mites including *A. drabae* [J. Littlefield, unpubl. data] and *F. perrepae* [125]. An alternative would be to inoculate plants using small pieces of gall or plant material that have been inspected and cleaned of other organisms [142,144]. Rearing colonies are sometimes established with limited numbers of mites due to low populations found during overseas collections, shipping mortality, and limited rearing space in containment facilities [146]. These containment rearing colonies serve as sources of mites for initial field release. Since containment colonies are generally unnecessary once the agent becomes established, little emphasis has been placed on efficient rearing techniques. For the augmentations of *A. malherbae* in Mexico, Rodriguez-Navarro et al. [147,148] did investigate inoculation levels and quality standards (number of mites per gall, number of galls per plants, infested stems, etc.) required for the successful laboratory rearing of a biocontrol agent over multiple generations without loss of genetic diversity [149,150].

The majority of eriophyid mites utilized for biological control of weeds are gall-makings, leaf distorters or rust mites. Release techniques to establish eriophyid mites have primarily relied on distributing galls or infested plant tissues or transplanting infested plants. Techniques may vary depending upon the intent of the release, e.g., initial release of a mite from containment with the goal of establishing populations or post–establishment redistribution either within site or to other locations. For example, the *A. malherbae* initial releases in Texas, USA [143], were made by inoculating potted plants and then transferring these to field plots. In Montana, USA, and Alberta, Canada [144], *A. malherbae* was released using two methods: either infested field bindweed plants from greenhouse rearing colonies were transplanted to field sites or infested leaves or stems were distributed at sites. Infested material was held next to healthy plants by twist ties or with Parafilm^®^ or sections of split plastic straws. Both techniques were successful in Montana. Using infested plant material requires that there be close contact between infested and healthy plants. Friend et al. [151] reported that establishment of *A. malherbae* in Oklahoma, USA, was limited when infested material was simply placed on healthy plants. Wrapping infested stems around healthy stems improved successful transfer of the mite.

Once mites are established, they are often very effectively dispersed to new locations via wind. For example, *A. malherbae* in Montana was first established in the mid-1990s [144], and a follow-up survey conducted in 2007 showed that the mite was well dispersed across much of eastern Montana [152]. *Aceria genistae* first appeared as an adventive in North America in Tacoma, Washington, and Portland, Oregon, in 2005 [153]. In 2014, it was detected in California, at Georgetown and in the Sierra Nevada foothills, which is at least 760 km away. Pratt et al. [153] estimated that long range dispersal from Washington to California ranged from 39 to 62 km/yr. However, the fact that Sierra Nevada foothills were colonized by *A. genistae* prior to areas along the state’s northern border or coast suggests that human-facilitated movement, such as by the movement of motorized vehicles or timber equipment may have been important. In any case, by 2017 the mite occurred over a region about 1500 km wide and at elevations ranging from 18 to 1160 m above sea level.

Post-release monitoring is generally designed to detect establishment, population density and dispersal of the agents; the degree of infestation or attack on target plants at the individual and/or population level; and possible impacts or interactions between biological control agents and nontarget organisms and/or critical habitats [116,154]. Biocontrol monitoring activities typically involve sampling arthropod populations along with target plant populations and the associated plant community. Monitoring protocols vary with the specific biocontrol agent and target plant [155,156]. Eriophyid populations may be measured directly by extracting mites from infested plant material [157,158,159]. The presence and dispersal of eriophyid mites in the field may also be detected by utilizing various traps including water pan traps, sticky traps or slides coated with silicone grease or petroleum jelly, or modified spore collection devices [86,97,160]. Indirect methods, such as counting galls or recording plant damage, are often employed due to ease and economy of sampling. Counts may be made on a per unit basis, either by area or by number of plants sampled, or on a timed basis. Accuracy of such counts may be dependent upon eriophyid population development, timing of samples, plant phenology, etc. More recently, DNA finger printing has been employed to determine genetic shifts with expanding mite populations and to track specific eriophyid genotypes/haplotypes, such as for the European broom gall mite, *A. genistae*, which is adventive in western USA [153]. Funding for release and post-release monitoring of agents is often limited and frequently these activities are strictly aimed at the evaluation of the outcomes (e.g., agent establishment and impact) in term of management of the invasive weed. Schaffner et al. [116] suggested that an ecological approach to agent monitoring should also be investigated to predict and detect changes to ecosystem processes and services (e.g., food production, human health, tourism/recreation) brought about by biological control. They suggest that post-release monitoring can advance classical biological control of weeds by testing predictions or hypotheses derived from pre-release studies thus making monitoring more ecological and holistic in scope.

## 7. Conclusions and Recommendations

The importance of eriophyid mites, whether they are pests or biological control agents, is mainly due to the damage they can inflict on their hosts. These tiny plant feeders possess several of the desirable features for a biological control agent; however, in order to use them in weed classical biological control programs some important challenges must be overcome. Proper taxonomic identification of the eriophyid mite candidate is one of these. Nowadays, the use of advanced microscopic methods and molecular genetic tools, as well as studies of eriophyid biology, ecology, and behavior, is improving the description and characterization of eriophyid taxa. This modern approach to the taxonomy can facilitate investigations regarding eriophyid mites in general, and their interactions with the host plant, and consequently also their potential use as classical biological control agents of invasive weeds.

The growing demand in making biological control programs as safe as possible, and hence prevent any potential negative effect due to the release of the agent, imposes a deep understanding of the host range of the candidate and an accurate risk assessment for nontarget organisms. The achievement of these aims cannot ignore, especially in the case of eriophyid mites, the use of a combined approach of both laboratory and field tests. Moreover, the evaluation of the risk to nontarget plants cannot either overlook survival, reproduction, and development of the mite candidate on various host plants, or its ability to damage them.

The safety of the biological control agent released is usually considered a priority and has always been emphasized by regulatory agencies. However, characterization of impacts on the target and potential establishment in the introduced environment are just as important to the success of a classical biological control program, and more efforts and funds should be focused on these aspects.

Impact studies can be made particularly challenging by the phenomena of host-adapted mite strains or variability in the susceptibility of the plant to the mite. Therefore, genetic studies of both agent and target are crucial to approach properly this challenge. Moreover, it is important to consider eriophyid biology and ecology, as well as that of the target weed, and potential biotic and abiotic limiting factors before developing an experimental design and deciding how and which parameters to measure. An in-depth analysis of the potential limiting factors that could negatively affect the establishment and hence also the efficacy of the agent, can also support the release strategies and hence increase its potential success.

The final steps of release and post-release monitoring should not be underestimated, and the methods to accomplish them should be chosen with care according to the primary goals of the program. Post-hoc analyses of these steps are advantageous to improve the release strategies and favor the establishment of the agents, and hence increase the general success of classical biological programs. In particular, by the analysis of the results obtained by post-release monitoring activities, it is possible to understand if all challenges here mentioned have been successfully addressed.

Finally, it is important to highlight that, although eriophyid mites were first noted in the literature about 270 years ago and have been extensively investigated since the mid-19th century, studies on their biology, ecology and behavior have only been undertaken for a few decades, and rather sparsely. A long list of unresolved questions could be proposed, such as regarding their life history and physiological adaptation (e.g., survival strategies in relation to the host plant species, and to environmental conditions), or the interactive mechanisms between them and their hosts (e.g., biochemistry of mite-plant relationships; attractive, acceptance and repellence mechanisms of the host plant; possible co-evolution and speciation phenomena). Biological control programs would definitely benefit from this knowledge, which would help improve the design of the experiments to test host specificity, assess the risk of nontarget species, and evaluate the impact on the target. It would also help improve interpretation of the results obtained, and guide practical aspects directly connected with putting in place a biological control program and improve its general success. For example, the knowledge of the mechanisms and factors influencing the dispersal of eriophyid mites is of great importance for managing and monitoring their release, but also for predicting their success in colonization and establishment.

In conclusion, although eriophyid mites seem to have what it takes to become one of the best groups of biological control agents, it is clear that to attain this status scientists need to learn how to better deal with them, starting from increasing the knowledge of their basic biology, ecology and behavior. Unfortunately for eriophyid mites, one size does not fit all, and each individual species or system will have its own challenges, however, the potential benefits do out weight the costs.

## Figures and Tables

**Table 1:** Native or exotic eriophyid mite species intentionally redistributed or intentionally introduced (i.e., released), respectively; exotic species adventive or officially permitted as biological control agents against invasive weeds. Ecological class (i.e., gall-making or vagrant), target species, area of action, year of record, establishment, level of impact and potential limiting factors to the efficacy of the agent are reported for each eriophyid mite species listed. Data are from 1968 up to today, adapted from Smith et al. [23], Weyl et al. [26], Winston et al. [27,28], and references therein.

Eriophyid Mite Species	Ecological Class	Target Species	Status	Region	Location	Year	Establishment	Impact	Limiting Factors
*Acalitus adoratus* Keifer	gall- making	*Chromolaena odorata* (L.) R.M. King & H. Rob. (Asteraceae; Saimweed)	Adventive	Asia	Bangladesh	2009	Yes	Slight	-
					China	1991	Yes	Slight	-
					India	2005	Yes	Slight	-
					Indonesia	1991	Yes	Slight	-
					Laos	2009	Yes	Slight	-
					Malaysia	1970s	Yes	Slight	-
					Myanmar	2009	Yes	Slight	-
					Philippines	1987	Yes	Slight	-
					Singapore	2009	Yes	Slight	-
					Taiwan	1992	Yes	Slight	-
					Thailand	1984	Yes	Slight	-
					Timor-Leste	2003	Yes	Slight	-
					Vietnam	2009	Yes	Slight	-
				Pacific	Guam	2005	Yes	Slight	-
					Micronesia	1988	Yes	Slight	-
					Northern Marianas	2005	Yes	Slight	-
					Palau	1998	Yes	Slight	-
					Papua New Guinea	2005	Yes	Slight	-
*Aceria acroptiloni* Shevchenko & Kovalev	gall- making	*Rhaponticum repens* (L.) Hidalgo (Asteraceae; Russian knapweed)	Intentionally redistributed	Asia	Uzbekistan	1997	Yes	Heavy	-
				Eurasia	Ukraine	1973	Yes	Heavy	-
*Aceria angustifoliae* Denizhan et al.	gall- making	*Elaeagnus angustifolia* L. (Elaeagnaceae; Russian olive)	Petition submitted ^1^	North America	Canada	2019	-	-	-
					USA	2019	-	-	-
*Aceria anthocoptes* (Nalepa)	vagrant ^2^	*Cirsium arvense* (L.) Scop. (Asteraceae; Canada thistle)	Adventive	North America	Canada	2011	Yes	Unknown	-
					USA	1998	Yes	Slight	-
*Aceria chondrillae* (Canestrini)	gall- making	*Chondrilla juncea* L. (Asteraceae; rush skeletonweed)	Adventive	North America	Canada	1993	Yes	Slight	-
			Intentionally introduced	Australia	Australia	1971	Yes	Variable	Specificity Climate
						1985	Yes	Slight	-
				North America	USA	1977	Yes	Variable	Predation Specificity Climate
				South America	Argentina	1989	Yes	Unknown	-
*Aceria davidmansoni* Xue, Han & Zhang	gall- making	*Ulex europaeus* L. (Fabaceae; gorse)	Adventive	Pacific	New Zealand	1985	Yes	Slight	-
*Aceria drabae* (Nalepa)	gall- making	*Lepidium draba* L. (Brassicaceae; hoary cress)	Intentionally introduced	North America	USA	2019	Too early ^3^	Too early	-
*Aceria genistae* (Nalepa)	gall- making	*Cytisus scoparius* (L.) Link (Fabaceae; Scotch brrom)	Adventive	North America	Canada	2007	Yes	Slight	-
					USA	2005	Yes	Variable	Possibly predation
			Intentionally introduced	Australia	Australia	2008	Yes	Unknown	-
				Pacific	New Zealand	2007	Yes	Variable	Specificity Possibly predation
*Aceria lantanae* (Cook)	gall- making	*Lantana camara* L. *sens. lat.* (Verbenaceae; lanatana)	Adventive	Africa	Kingdom of Eswatini	2010	Yes	Unknown	-
					Malawi	2019	Yes	Variable	Possibly specificity
					Mozambique	2017	Yes	Unknown	-
					Zambia	2019	Yes	Variable	Possibly specificity
			Intentionally introduced	Africa	South Africa	2007	Yes	Variable	Host plant resistance Climate
				Australia	Australia	2012	Yes	Variable	Possibly predation Specificity
				North America	USA	1976	Yes	Unknown	-
*Aceria malherbae* Nuzzaci	gall- making	*Convolvulus arvensis* L. (Convolvulaceae; field bindweed)	Intentionally introduced	Africa	South Africa	1994	No	-	Land use
				North America	Canada	1989	Yes	Unknown	Climate
					Mexico	2004	No	-	-
					USA	1989	Yes	Variable	Possibly host plant resistance Climate
		*Calystegia sepium* (L.) R. Br. (Convolvulaceae; hedge bindweed)	Intentionally introduced	North America	USA	1993	Unknown	Unknown	-
*Aceria salsolae* de Lillo & Sobhian	vagrant ^2^	*Salsola tragus* L. (Chenopodiaceae; Russian thistle)	Petition submitted ^4^	North America	USA	2004	-	-	-
*Aceria* sp.	gall- making	*Chrysanthemoides monilifera* (L.) Norl. subsp. *monilifera* (Asteraceae; boneseed)	Intentionally introduced	Australia	Australia	2008	Yes	Slight	Possibly predation Climate
*Aceria vitalbae* (Canestrini)	gall- making	*Clematis vitalba* L. (Ranunculaceae; old-man’s beard)	Petition approved ^5^	Pacific	New Zealand	2018	-	-	-
*Aculus crassulae* Knihinicki & Petanović	gall- making	*Crassula helmsii* (Kirk) Cockayne (Crassulaceae; swamp stonecrop)	Intentionally introduced ^6^	Eurasia	UK	2018	Too early ^7^	Too early	-
*Aculus hyperici* (Liro)	vagrant	*Hypericum perforatum* L. (Hypericaceae; St John’s wort)	Intentionally introduced	Australia	Australia	1985	No	-	Possibly predation Possibly parasitism
						1991	Yes	Slight	Host plant resistance
*Cecidophyes rouhollah* Craemer	gall- making	*Galium aparine* L. (Rubiaceae; cleavers)	Adventive	Pacific	New Zealand	2017	Yes	Unknown	Possibly predation
			Intentionally introduced	North America	Canada	2003	No	-	Climate
*Colomerus spathodeae* (Carmona)	gall- making	*Spathodea campanulata* P. Beauv. (Bignoniaceae; African tulip tree)	Adventive	Africa	Malawi	2019	Yes	Unknown	-
			Intentionally introduced ^8^	Pacific	Cook Islands	2017	Yes	Unknown	-
*Floracarus perrepae* Knihinicki & Boczek	gall- making	*Lygodium microphyllum* (Cav.) R. Br. (Lygodiaceae; Old World climbing fern)	Intentionally introduced	North America	USA	2008	Yes	Variable	Specificity
*Phyllocoptes* *fructiphilus* Keifer	gall- making	*Rosa multiflora* Thunb. (Rosaceae; miltiflora rose)	Adventive	North America	USA	1968	Yes	Variable	Plant stage

^1^ Petition for the field release of *Aceria angustifoliae* was submitted to the U.S Department of Agriculture, Animal Plant Health Inspection Service, Technical Advisory Group (USDA APHIS TAG) and the Canadian Biological Control Review Committee (BCRC) in November 2019. Canadian Food Inspection Agency (CFIA) denied a permit on 20 May 2020, despite the BCRC recommending release. TAG recommended approval on 27 May 2020, whereas APHIS’s decision is still pending [29,30]; ^2^ It can also cause deformations or galls; ^3^ Even though it is too early to evaluate the establishment of *Aceria drabae*, mites were able to overwinter and in 2020 they were recorded at the sites of release [J. Littlefield, unpubl. data]; ^4^ TAG recommended approval on 8 August 2005, however APHIS denied a permit on 14 May 2009 [31]; ^5^ Approval of the field release of *Aceria vitalbae* in New Zealand was gained in October 2018 [32]. A small population of *A. vitalbae* on potted *Clematis vitalba* was removed from containment in December 2019 to increase mite numbers prior to field release. In spring 2020, mite numbers were assessed as low, thus field releases were postponed. Currently, mite rearing on potted plants held in shade-houses is continuing with first field releases of *A. vitalbae* in heavily infested *C. vitalba* areas planned for early next spring (August/September 2021) [33]; ^6^ Approval the field release of *Aculus crassulae* in England and Wales was gained in summer 2018 [34], but only a small release was carried out before the end of the same year (September 2018). Main releases took place from 2019 [35]; ^7^ Even though it is too early to evaluate the establishment of *Aculus crassulae*, mites were able to overwinter, and in 2020 they were recorded at a couple of sites in the release areas but in low numbers. However, the potential of *A. crassulae* establishment under the UK climate was investigated before of the release, and it was shown that the mite would be able to complete several generations during the UK growing period, with more generations possible in southern parts of the UK [35]; ^8^ [36].

## Data Availability

Not applicable.
